# Perfoliate leaves reduce herbivory in the shield‐bracted monkeyflower ( *Mimulus glaucescens* )

**DOI:** 10.1002/ecy.3876

**Published:** 2022-11-27

**Authors:** Katherine Toll

**Affiliations:** ^1^ Department of Plant Biology Michigan State University East Lansing Michigan USA

**Keywords:** herbivory, monkeyflower, perfoliate

Many plant species have leaves that are completely fused around the stem, forming a disk or cup, a trait called perfoliation (Appendix S1: Figure S1). This morphology has long fascinated naturalists (Beal & St. John, 1887; Christy, 1923; Darwin, 1789, 1877a, 1877b; Keller, 1987). Much of this interest has been in plants with cuplike fused leaves that collect water (e.g., teasel, *Dipsacus*, cup plant, *Silphium*). Ecological hypotheses to explain the presence of perfoliate leaves include water reservoirs for periods of drought, plant carnivory via insect capture, and preventing herbivore movement via the moat that forms around the stem (Beal & St. John, 1887; Carlson & Harms, 2007; Darwin, 1789, 1877a, 1877b; Krupa & Thomas, 2019; Shaw & Shackleton, 2011; Sun & Huang, 2015). Despite the interest, these hypotheses are, at best, weakly supported (Krupa & Thomas, 2019), suggesting that water capture may be an incidental occurrence rather than an adaptive function. Further, most plants with perfoliate leaves do not collect water, though these surfaces might still restrict or prevent insect herbivore movement up and down the plant stem. I became interested in this while observing caterpillars interacting with a variety of California monkeyflowers (*Mimulus*).

The aptly named shield‐bracted monkeyflower, *Mimulus glaucescens* (syn. *Erythranthe glaucescens*), produces two leaf types: round to ovate basal petiolate leaves (hereafter basal leaves) and distinct inflorescence leaves formed after individual plants transition to flowering that completely fuse around the stem, forming round disks (hereafter perfoliate bracts, Figure 1). In several populations, I noticed that the basal leaves had heavy feeding damage, whereas the perfoliate bracts had little to no damage (Figure 1). *M. glaucescens* often co‐occurs with other closely related monkeyflowers in the *Mimulus guttatus* species complex with very similar morphologies besides these specialized leaves. While at a site where *M. glaucescens* co‐occurs with *Mimulus nasutus*, I also noticed differences in the distribution of feeding damage between species.

**FIGURE 1:**
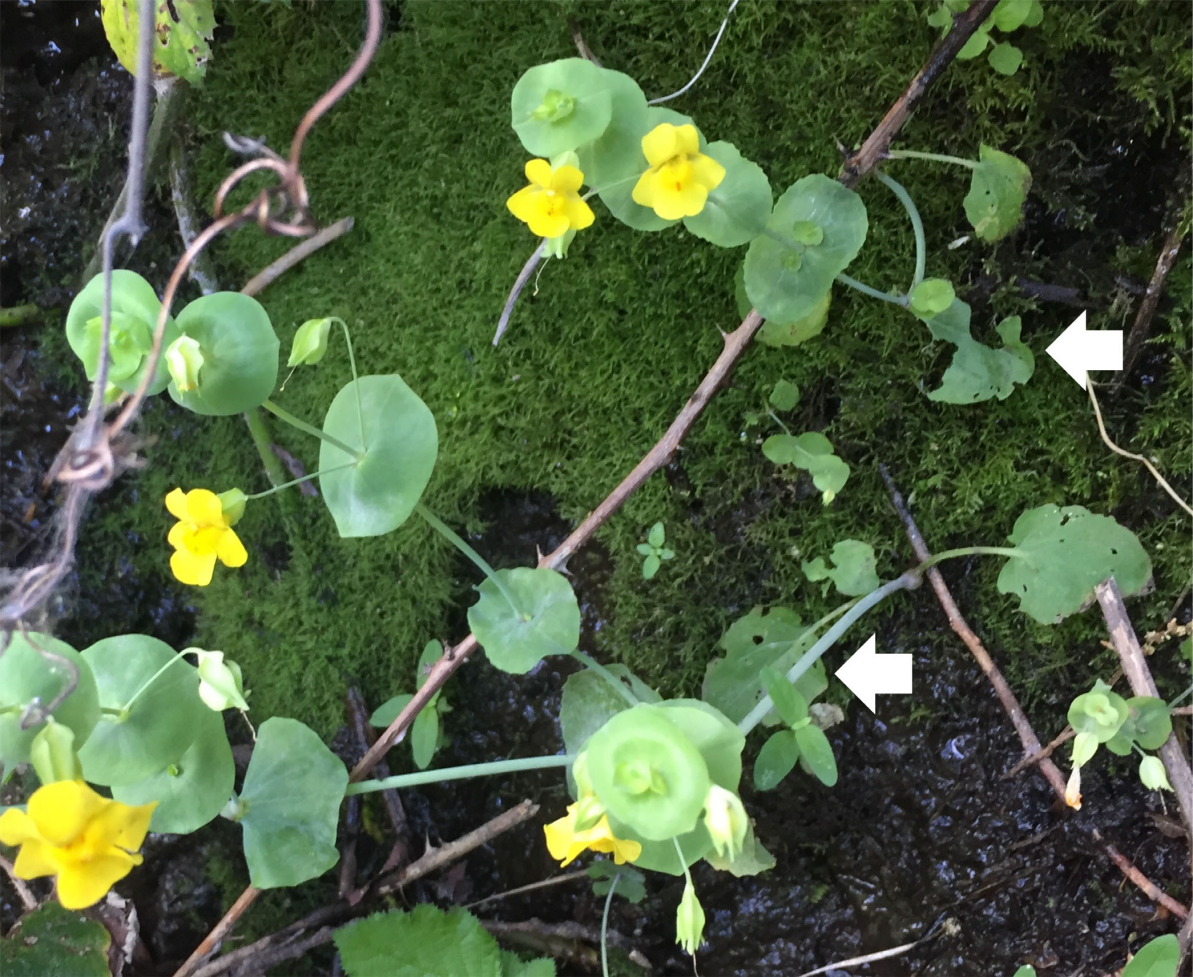
*Mimulus glaucescens* observed at 2:00 PM on 6 June 2018 on Honey Run Road in Paradise, CA (39.74970° N, 121.64067° W). White arrows indicate basal leaves with herbivore damage. Photo by K. Toll.

To quantify the relative amount of damage received by perfoliate bracts versus basal leaves, I surveyed 24–34 randomly chosen plants among five populations of *M. glaucescens* (locations in Appendix S1: Table S1). On every plant, at each node (i.e., a pair of perfoliate bracts or basal leaves), I recorded the presence or absence of herbivore damage (0 or 1), the type of leaf (perfoliate or basal) and the position of the node (the first true leaf pair = 1, second leaf pair = 2, and so on). To test whether perfoliate and basal leaf pairs differed in the probability of damage, I fit a binomial mixed model with damage as the dependent variable, leaf type and population as independent variables, and individual plant ID as a random effect. All mixed models were fit using the lme4 package (version 1.1.28, Bates et al., 2015) in R (version 4.1.2, R Core Team, 2021). The probability of damage and 95% confidence intervals were predicted from mixed models using the R package ggeffects (version 1.1.1, Lüdecke, 2018).

Consistent with my observations, perfoliate bracts had far less damage than basal petiolate leaves across all five populations (Figure 2). Leaf type and population were significantly associated with the probability of herbivore damage (Wald type II χ^2^ test: leaf type χ^2^ = 203.787, df = 1, *p* < 0.001; population χ^2^ = 14.629, df = 4, *p* = 0.006). Transforming the binomial coefficient, the model predicted that basal leaves were 59% to 103% more likely to be damaged than perfoliate bracts across all populations (Figure 2). Although these results strongly demonstrate a difference in herbivory, the underlying cause might not be the perfoliation, per se.

**FIGURE 2:**
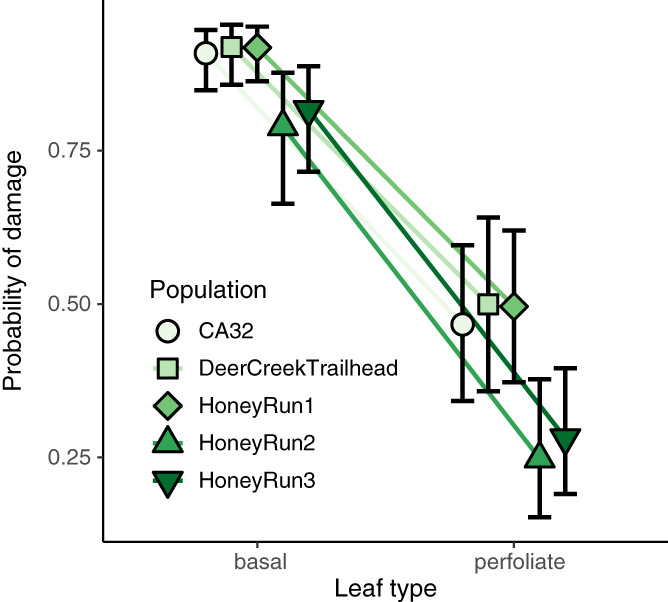
Perfoliate bracts are less likely to receive herbivore damage than basal leaves in five populations of *Mimulus glaucescens*. The probability of damage and 95% confidence intervals were predicted from a binomial model mixed model.

Perfoliate bracts are only produced after a plant has transitioned to flowering. Thus, the difference in the probability of damage between perfoliate bracts and basal leaves might simply be due to a difference in the timing of the production of different leaf types; the later perfoliate bracts may just have had less time to accrue damage. Other changes associated with phenology could also result in the same pattern. For instance, the production of secondary metabolites within the plant or the herbivore community may have changed over time. At one of the censused populations (Deer Creek Trailhead 40.17306° N, 121.55608° W), *M. glaucescens* co‐occurred both spatially and phenologically with a closely related species, *M. nasutus*, that only produces ovate petiolate leaves, both before and during flowering. Therefore, if most herbivory occurred early in the season and that underlay the previous difference, we would expect a decline in both species with increasing nodes (a proxy for time, independent of perfoliation). Using the same methodology as the previous surveys, I also censused 30 *M. nasutus* individuals. To test whether the probability of leaf pair damage changed with developmental time and whether this differed between species at this site, I fit a binomial mixed model with damage as the dependent variable and node, species, and their interaction as the independent variables, with individual plant as a random effect. I tested whether the slope of the relationship between node and damage significantly differed from 0 for each species using the R package emmeans version 1.7.2 (Lenth, 2022).

Damage gradually decreased with increasing nodes for *M. glaucescens* but not co‐occurring *M. nasutus* (Figure 3). The probability of herbivore damage was significantly associated with species (Wald type III chi‐square test: χ^2^ = 23.01, df = 1, *p* < 0.001), node (χ^2^ = 16.33, df = 1, *p* < 0.001), and their interaction (χ^2^ = 8.06, df = 1, *p* = 0.005). The probability of damage for *M. glaucescens* decreased from 0.89 for the first node to 0.53 for the 10th node. For *M. nasutus* there was no effect; the relationship between node and damage probability did not significantly differ from zero (slope = 0.012, SE = 0.06, *z*‐ratio = 0.21, *p* = 0.83). The difference in distribution of damage between species suggests that perfoliate leaves do not experience less damage because they are produced later since these two co‐occurring species share herbivores and bolt and flower at similar times at this single site. Perfoliation may not fully explain the difference in the distribution of damage between plants because other untested factors may have changed with flowering differently in each species. For example, *M. glaucescens* produces epicuticular leaf waxes upon flowering that may deter herbivores (Eigenbrode & Espelie, 1995), although these waxes are readily eroded by rainfall (Baker & Hunt, 1986); in contrast, *M. nasutus* does not produce obvious waxes.

**FIGURE 3:**
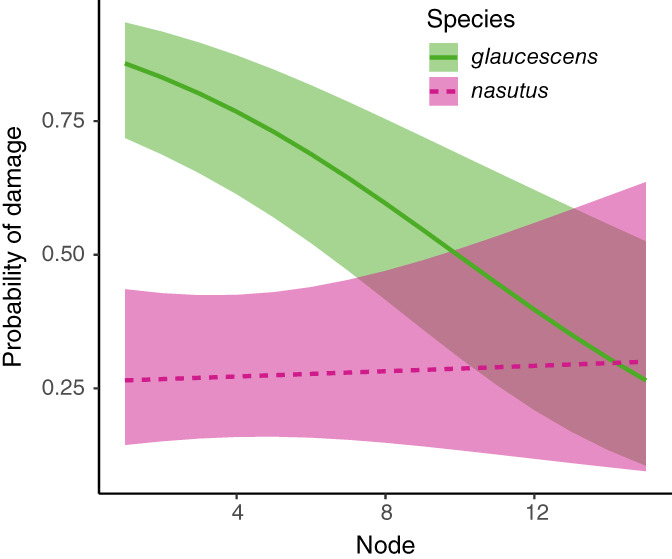
Damage decreased with increasing nodes for *Mimulus glaucescens* (green solid line) but not *M. nasutus* (pink dashed line) at a single site where these species co‐occurred. The probability of damage and 95% confidence intervals (shaded areas) were predicted from a binomial mixed model.

To determine whether perfoliate bracts delayed or reduced a crawling herbivore's ability to walk on the plant's surface, I collected and repotted 44 plants from two populations (CA32 and Honey Run 1; Appendix S1: Table S1) and herbivores in the field: 40 early‐instar variable checkerspot caterpillars (*Euphydryas chalcedona*). Variable checkerspots and a closely related species, the common buckeye (*Junonia coenia*), usually feed primarily on reproductive structures and spend much time on the apices of *Mimulus* plants (Toll & Willis, 2018; Appendix S1: Figure S2). I placed caterpillars at the base of each plant and recorded how much time it took them to reach a perfoliate bract, whether they passed or turned around at the first perfoliate bract, and whether they made it to the apex of the plant. Most checkerspot caterpillars were able to climb over the first perfoliate bract they encountered (19/23 caterpillars on CA32 plants and 16/17 caterpillars on HoneyRun1 plants), but few reached the apex of each plant (7/23 and 4/17 caterpillars on CA32 and HoneyRun1 plants, respectively). Although I did not have a formal comparison, given the observed behavior of these caterpillars on plants without perfoliate bracts in the field, their inability to reach the higher‐valued reproductive structures suggests that one ecological function of these bracts may be to impede herbivore movement.

How perfoliate bracts interact with different herbivores remains an open and intriguing question. Yellow monkeyflowers are attacked by a diversity of herbivores, including caterpillars (Levine, 1999), spittlebugs (Carr & Eubanks, 2002), leaf mining flies (Eiseman, 2019), leaf hoppers and grasshoppers (Elderd & Doak, 2006), and voles (Popovic & Lowry, 2020). I hypothesize that perfoliate bracts are unlikely to prevent damage from large or flighted herbivores and are likely most effective against small crawling herbivores, though this ought to be explicitly tested.

In many of the well‐known species with perfoliate leaves, including miner's lettuce (*Claytonia perfoliata*), perfoliate peppergrass (*Lepidium perfoliatum*), honeysuckles (*Lonicera sempervirens*), and many more (Appendix S1: Figure S1), the perfoliate leaves are directly below the inflorescences. The perfoliate leaves of *Claytonia perfoliata*, produced just before flowering, also receive less herbivore damage than nonperfoliate leaves, and wooly bear caterpillars prefer nonperfoliate leaves when given a choice (R. Karban, personal communication). My results suggest that these structures, which are usually formed after plants transition to flowering, though potentially involved in many functions, lessen damage to tissues most directly related to fitness (buds, flowers, and fruit), consistent with the optimal defense hypothesis (Rhoades, 1979). The totality of the varied data sets collected here provides a compelling explanation for a remarkable trait that has fascinated generations of naturalists. I hope these observations inspire investigation of more of these interesting species.

## CONFLICT OF INTEREST

The authors declare no conflict of interest.

## Supporting information


Appendix S1

## Data Availability

Data and code (Toll, 2022) are available in Figshare at https://doi.org/10.6084/m9.figshare.20280249.v1.
