# Supplementary material for: Perfoliate leaves reduce herbivory in the shield‐bracted monkeyflower ( Mimulus glaucescens )
Source: Ecology. 2022 Nov 27;104(1):e3876. doi: 10.1002/ecy.3876 (PMC10078401; doi:10.1002/ecy.3876)
Supplement: Supplementary file 1 — Appendix S1 [file ECY-104-0-s001.pdf]

Katherine Toll. Perfoliate leaves reduce herbivory in the shield-bracted monkeyflower (*Mimulus glaucescens*). *Ecology*.

Table S1. Location of populations surveyed and sample sizes of *M. glaucescens* surveyed at each site.

Population	Latitude	Longitude	n
CA32	40.12623° N	121.57240° W	34
DeerCreekTrailhead	40.17306° N	121.55608° W	25
HoneyRun1	39.75214° N	121.63668° W	32
HoneyRun2	39.74970° N	121.64067° W	24
HoneyRun3	39.74923° N	121.64267° W	34

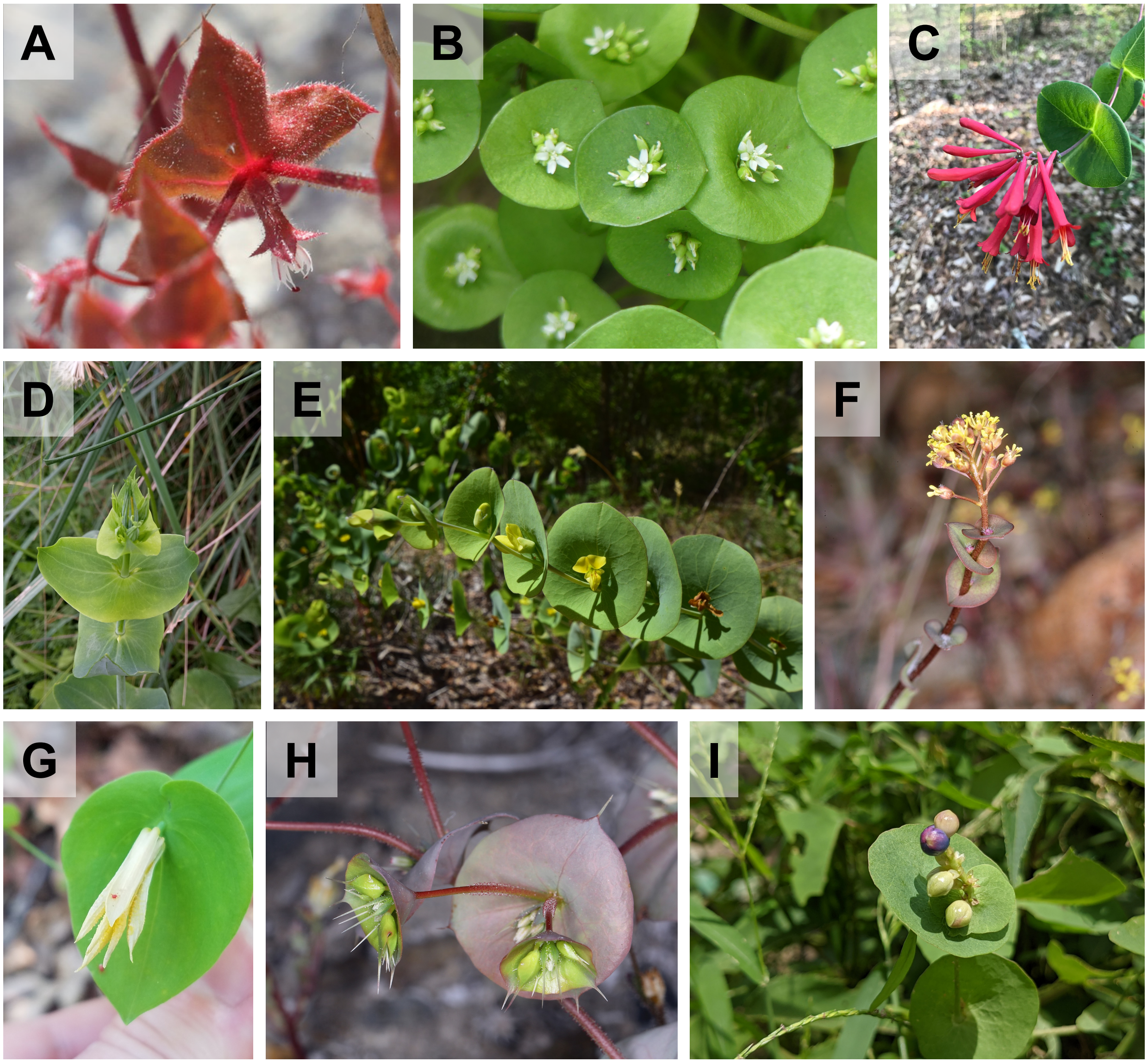

Figure S1. A sampling of species with perfoliate leaves. (A) *Mucronea perfoliata* (A. Gray) A. Heller (https://www.gbif.org/occurrence/1850928317) observed in United States of America by Paul G. Johnson (licensed under http://creativecommons.org/licenses/by-nc-sa/4.0/). (B) *Claytonia perfoliata* Donn. (https://www.gbif.org/occurrence/3743290627) observed in United States of America by Lauren Glevanik (licensed under http://creativecommons.org/licenses/by-nc/4.0/). (C) *Lonicera sempervirens* L. (https://www.gbif.org/occurrence/2597896983) observed in United States of America by Sarah (licensed under http://creativecommons.org/licenses/by/4.0/). (D) *Blackstonia perfoliata* (L.) Huds. (https://www.gbif.org/occurrence/3079682809) observed in Algeria by Karim Haddad (licensed under http://creativecommons.org/licenses/by/4.0/) (E) *Baptisia perfoliata* (L.) R. Br. (https://www.gbif.org/occurrence/2802906675) observed in United States of America by gman122 (licensed under http://creativecommons.org/licenses/by-nc/4.0/). (F) *Lepidium perfoliatum* L. (https://www.gbif.org/occurrence/3455518822) observed in United States of America by Steve Matson (licensed under http://creativecommons.org/licenses/by-nc/4.0/). (G) *Uvularia perfoliata* L. (https://www.gbif.org/occurrence/2597861256) observed in United States

of America by Leila Dasher (licensed under http://creativecommons.org/licenses/by/4.0/). (H) *Oxytheca perfoliata* Torr. & A. Gray (https://www.gbif.org/occurrence/3784827902) observed in United States of America by Steve Matson (licensed under http://creativecommons.org/licenses/by-nc/4.0/). (I) *Persicaria perfoliata* (L.) H. Gross (https://www.gbif.org/occurrence/3124705266) observed in Chinese Taipei by 長鬃山羊 (licensed under http://creativecommons.org/licenses/by-nc/4.0/).

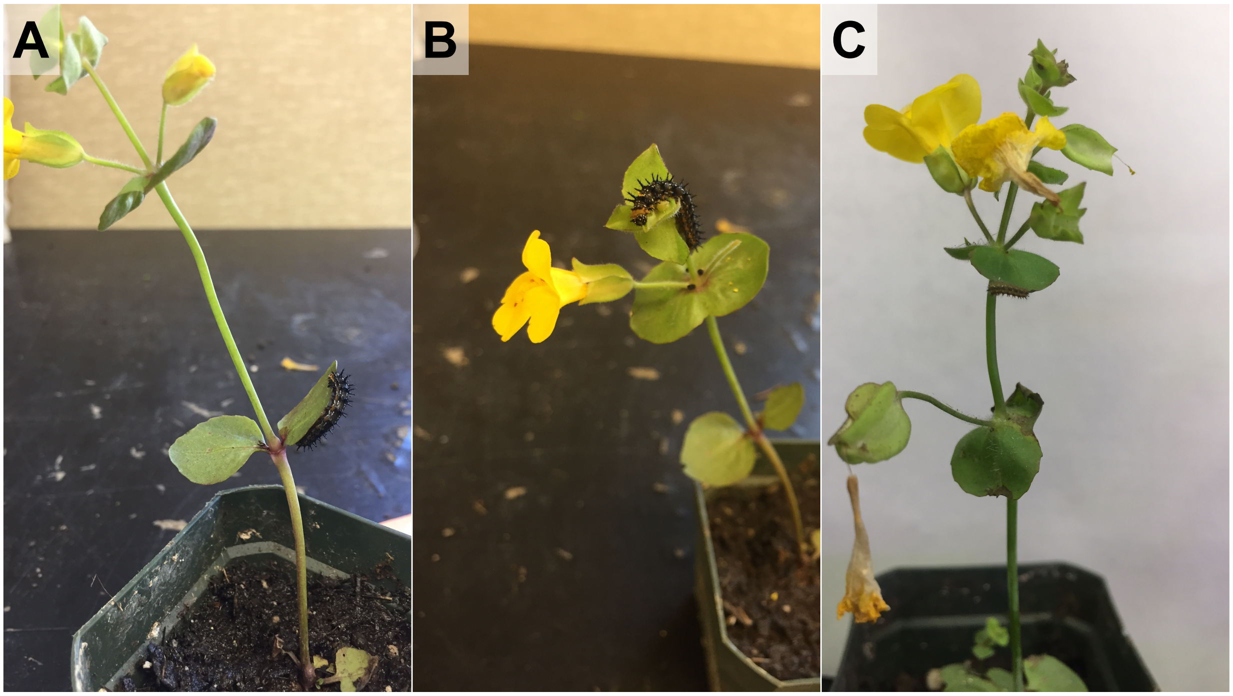

Figure S2. Observations of crawling herbivore movement on field collected plants. Due to low sample sizes of buckeyes, I only reported behavior of variable checkerspot caterpillars. (A) A buckeye caterpillar (*Junonia coenia*) chewing on a basal leaf of *M. glaucescens*, (B) A buckeye caterpillar (*Junonia coenia*) at the apex of an *M. glaucescens* plant. (C) A variable checkerspot caterpillar (*Euphydryas chalcedona*) under a perfoliate bract of *M. glaucescens*.
